# Therapeutic Anticoagulant Does not Modify Thromboses Rate Vein after Venous Reconstruction Following Pancreaticoduodenectomy

**DOI:** 10.1155/2008/896320

**Published:** 2008-11-23

**Authors:** Mehdi Ouaïssi, Igor Sielezneff, Nicolas Pirro, Rémi Bon Mardion, Jean Batiste Chaix, Abdelrhame Merad, Stéphane Berdah, Vincent Moutardier, Silvia Cresti, Olivier Emungania, Loundou Anderson, Brunet Christian, Sastre Bernard

**Affiliations:** ^1^Service de Chirurgie Digestive et Oncologique, Pôle d'Oncologie et de Spécialité Médicale et Chirurgicale, Hôpital Timone, 13385 Marseille, France; ^2^Faculté de Médecine, Université d'Aix Marseille II, 13284 Marseille, France; ^3^Service de Chirurgie Digestive, Hôpital Nord, 13915 Marseille, France; ^4^Service de Santé Publique et Epidémiologie, Faculté de Médecine de la Timone, Hôpital Timone, 13385 Marseille, France

## Abstract

Recommendations for anticoagulation following major venous reconstruction for pancreatic adenocarcinoma (PA) are not clearly established. The aim of our study was to find out the relation between postoperative anticoagulant treatment and thrombosis rate after portal venous resection. *Materials and methods*. Between 1986 and 2006, twenty seven portal vein resections were performed associated with pancreaticoduodenectomies (*n* = 27) (PD).We defined four types of venous resection: type I was performed 1 cm above the confluent of the superior mesenteric vein (SMV) (*n* = 12); type II lateral resection and venorrhaphy at the level of the confluent SMV (*n* = 12); type III (*n* = 1) resulted from a primary end-to-end anastomosis above confluent and PTFE graph was used for reconstruction for type IV (*n* = 2). Curative anticoagulant treatment was always indicated after type IV (*n* = 2) resection, and after resection of type II when the length of venous resection was longer than ≥2 cm. *Results*. Venous thrombosis rate reached: 0%, 41%, and 100% for type I, II, IV resections, respectively. Among them four patients received curative anticoagulant treatment. *Conclusion.* After a portal vein resection was achieved in the course of a PD, curative postoperative anticoagulation does not prevent efficiently the onset of thrombosis.

## 1. INTRODUCTION

Resection of pancreatic duct adenocarcinoma has a high mortality rate [1]. Surgery still remains the only curative treatment, and tumoral invasion of the portal vein is not a contraindication of resection. Several techniques of venous resection and reconstruction have been described, such as primary lateral resection and venorrhaphy, primary circumferential resection with end-to-end anastomosis or graft interposition [2]. Recommendations for anticoagulation following major venous reconstruction for malignancy are not clearly established.

The aim of our study was to find out a relation between postoperative anticoagulant treatment and thrombosis portal rate after mesentericoportal segmental venous resection.

## 2. MATERIALS AND METHODS

From January 1996 to December 2006, 500 pancreatic resections were performed with curative intents in two surgical digestive departments. Twenty seven portal vein resections were performed in the course of a pancreaticoduodenectomy (PD). Clinicopathologic findings were analyzed to determine factors which are able to affect rates of morbidity and thrombosis in relation with venous reconstruction and anticoagulation treatment.

### 2.1. Operative technique

Contrast-enhanced computed tomography has been the most useful imaging exams to determine local respectability [3, 4]. When adherence to the lateral or posterior wall of the portal vein was encountered, surgeons preferred venous resection.

### 2.2. Pancreatic resection

Dissection of retroperitoneal margin was carried out prior to the venous resection. Aims of this technical choice were to avoid the need of venous anastomosis prior to the removal of the specimen to limit duration of venous occlusion and to allow as far as possible a preservation of the splenic vein. This was done either by a large kocher manoeuvre or by isolation of the superior mesenteric artery (SMA) both at its origin and the uncinate process. Kocher's manoeuvre oriented the SMA posterior to the portal vein and superior venous mesenteric and allowed an easy access for completion of the retroperitoneal dissection. Arterial branches coursing into the uncinate were sequentially clamped, divided, and ligated. Complete liberation of the pancreas from the SMA was done in the early course of the operation. The pancreatic head was then rotated back to its normal orientation; venous resection was performed as the final step of the resection.

### 2.3. Resection and venous reconstruction

Vascular control was always obtained proximally and distally taking care to isolate the superior mesenteric vein (SMV), portal vein (PV), and splenic vein (SV). Control of the gastric vein was sometimes required. We did not perform SMA occlusion because the duration of the clamping period of the PV was always less than 20 minutes. Type of resection is summarized in Table 1. A wedge resection was performed in case of limited ingrowths; elsewhere segmental resection was achieved when venous ingrowths appeared to be more extensive.

We defined four types of venous resection: (1) in type I, lateral resection and venorrhaphy of portal were performed; these resections were realized >1 cm above the SMV confluent (*n* = 12); (2) in type II, lateral resection and venorrhaphy of SMV and PV confluent were performed (*n* = 12); (3) in type III, a primary end-to-end anastomosis above the SMV and PV confluent was performed (*n* = 1); (4) in type IV, a circumferential resection (length >3 cm) of the SMV and PV confluent was carried out and PTFE graph was used for reconstruction (*n* = 2). Reimplantation of SV was never achieved in case of type IV resection.

### 2.4. Anticoagulant treatment

Curative anticoagulant treatment was always indicated for type IV (*n* = 2) resections and in four cases (30%) of type II resections when the length of venous resection was ≥2 cm. Curative anticoagulant was based on systemic anticoagulation therapy by heparin systemic anticoagulation therapy by Heparin (500 unit/kg/day) was indicated for 10 days in order to increase the activated partial thromboplastin time to 1.5–2.0 times that of control and then the treatment was discontinued at day 8. The oral anticoagulation drug (with antivitamine K) was given after control CT scan at day 8 to raise the mean international normalization ratio (INR). The rate of INR was between 2.5 and 3 and the treatment was performed during three months. Other patients had preventive anticoagulation by low molecular weight heparin.

### 2.5. Clinical and radiological follow up

All patients benefited from angiographic CT scan on day 7. Diagnosis of venous occlusion was settled on clinical findings (hepatic failure, fever), on biological data showing biological hepatic tests disturbances, and on CT scan findings. In the distant postoperative course, patients were followed clinically and by CT scan at 3, 6, and 12 months.

Post operative hospital stay and early outcome, were assessed. Surgical and/or medical complications were registered and classified as either minor or major. Postoperative pancreatic fistula was defined such as an international study group definition [5]. All pancreatic fistulas were deemed to be a major surgical complication. Biliary fistula was defined as biliary staining from drainage fluid. Pancreatic and/or biliary fistulas were treated by octreotide and antibiotics administration, or surgery if required. Postoperative gastric atony was defined by the need of inserting a nasogastric tube over day 4 or as delayed oral food intake after postoperative day 8. Mortality was defined as death occurring in the course of the hospitalization or within 3 months after discharge.

### 2.6. Study end point

Primary end points included postoperative survival, postoperative complications, and length of hospital stay.

### 2.7. Statistical analysis

Statistical analyses were conducted using SPSS version 13.0.1 (SPSS Inc, Ill, USA). Association between qualitative variables was assessed with chi-square test (or Fisher exact test, as appropriate). Means or median of continuous variables were analyzed by Student's *t*-test. Results of parametric and nonparametric data were expressed as mean (±SD) and median (±SD), respectively. All variables were dichotomized. Confidence intervals were set at 95%. Actuarial survival was analyzed by Kaplan Meier method and the survival difference was compared by log rang test. A value for *P* < .05 was considered to be statistically significant.

## 3. RESULTS

Between 1996 and 2006, 27 patients underwent portal venous resection in the course of a pancreatic resection for tumor. There were 15 males; mean age was 66 ± 9 (45–81) years old. Most of the patients were operated on for malignant tumors, pancreatic duct carcinoma (*n* = 25), and cholangiocarcinoma (*n* = 1), and in one case only for a pseudotumoral chronic pancreatitis (*n* = 1). Twenty seven PDs were performed. Preoperative risks were evaluated using the American Society Association (ASA) score. Most of the patients were ASA I or II (*n* = 11, *n* = 13), others were ASA score III (*n* = 3). Twelve patients had preoperative endoscopic bilary derivation.

Thirteen (48%) patients underwent a pylorus preserving PD, others (52%) underwent a standard PD. Mean time of the PD resection was 400 ± 108 minutes. Venous lateral resection was carried out using lateral venorrhaphy in 24 patients (89%); it was localized 1 cm above or on SMV confluent (*n* = 24), on the confluent (*n* = 12). In one case, we used autologous venous graph for a type II resection. For resections III and IV, primary end-to-end anastomosis, in one case, and PTFE graft were conducted twice. All types of resections are summarized in Table 1. Overall mortality was 11%. Overall morbidity was 13 (48%), including 7 (26%) cases of thrombosis, 4 (14%) cases of hemorrhagic complications, and one late pancreatic fistula successfully treated by radiological drainage. There were 5 (18%) cases of gastric atony. Sixteen (57%) patients required blood transfusion. The length of the hospitalization stay was 19 ± 9 days (Table 2).

### 3.1. Thrombosis complication

The most common perioperative complication was thrombosis which occurred in 7 patients (25%). It was diagnosed on clinical findings: all patients had fever, ascite, and mental troubles, on biological data: hepatic biological data increased 5 times the normal level in all the cases. CT scan was performed in 5 cases of thrombosis showing a total portal vein occlusion. Thrombosis occurred at mean day 4 and was associated to a hemoperitoneum in two cases. Surgical treatment consisted in thrombectomy and drainage of the hemoperitoneum. Despite this treatment, total portal vein thrombosis persisted and curative anticoagulation was continued for these two patients. One of them died after another surgical control with a new thrombectomy at day 18.

Three patients were successfully treated by curative anticoagulation. After six months, CT scan showed a persisting thrombosis implying the persistence of anticoagulation treatment. Two patients died of venous mesenteric infarction despite a surgical control at days 1 and 10. Among 7 cases of thrombosis, three deaths occurred after type II (*n* = 2) or type IV (*n* = 1) resection. Among them four patients received curative anticoagulant treatment. At the end of follow up, there was no more thrombosis or occluded anastomosis than the first cast.

### 3.2. Hemorrhage complication

Four patients had hemorrhagic complications requiring in all cases a surgical control. A portal vein thrombosis was associated twice with a hemorrhagic complication. Three patients received curative anticoagulant therapy. Surgical treatment of these hemorrhages needed lavage, drainage, and thrombectomy in case of portal thrombosis.

### 3.3. Long-term outcome

Mean of follow-up was 23 ± 31 months. Once the early postoperative course was achieved, no more cases of portal thrombosis occurred. There was no more thrombosis or occluded anastomosis than the first cast. Median disease-free survival was 16 months.

## 4. DISCUSSION

Although PD offers the only chance of cure for patients with adenocarcinoma of the pancreas, questions have arisen regarding the indication, safety, and outcomes of patients undergoing extended resections for locally advanced disease [6]. While a previous study demonstrated an overall survival benefit after pancreatic resection without an increase of morbidity and mortality rates [7–9], high mortality rate was reported for patients with portal vein thrombosis [10–13]. Few studies have focused on different types of surgical venous resections and different modes of reconstruction of the portal vein [6–14].

Few studies presented a radiological classification in order to predict the involvement of PV or SMV. Moreover, Nakao's report showed that macroscopic findings (classified into types A, B, C, or D according to preoperative findings on the portal phase of superior mesenteric angiography) were correlated with the histological invasion grades [15–17]. However, this classification was based on angiography or CT scan. In contrast, the necessity of curative anticoagulant therapy was evaluated by this preoperative classification. Moreover, our classification of resection and reconstruction permitted us to define the impact of resection of confluent and the modification of blood flow.

In this series, PD with venous resection was most commonly performed for pancreatic carcinoma. Most patients (89%) underwent a lateral venous resection with reconstruction by lateral venorrhaphy. Venous thrombosis rate reached 0%, 38%, and 100% for type I, II, and IV resections, respectively. One lateral venorrhaphy needed a patch of saphenous vein. Thrombosis occurred early after surgery, with an acute setting; no case of thrombosis appeared secondarily, at distance from the surgical removal of the tumor (after 6 months of follow-up). This result was in agreement with Leach's study which reported a thrombosis rate of 25% [10].

Moreover, in case of type II resection, patients with a portal resection length over 2 cm, or with PTFE reconstruction, received anticoagulation with intravenous heparin. In Leach's study, thrombosis rate raised to 25% despite the onset of a lasting treatment by low dose of aspirin [10].

This systematic onset of anticoagulation did not protect against venous thrombosis. These poor results were in accordance with Smoot's study which showed no difference in the rate of portal thrombosis between patients undergoing anticoagulant therapy and those without such treatment (*P* = .65) [6]. These results were confirmed in Carrere's study which demonstrated that only two cases of thrombosis occurred in patients under curative heparinotherapy [14]. Hemorrhagic complication increased after PD and anticoagulation. In Carrere's study, hemorrhage complications occurred after resection followed by postoperative curative anticoagulation. It required the immediate interruption of the anticoagulant therapy [14]. In our study, hemorrhage complications required 4 reinterventions. In fact, thrombosis appeared to be more affected by the type of reconstruction rather than by the anticoagulant treatment.

Lateral resection with lateral venorrhaphy on the confluent (SMV) must be avoided and replaced by a circumferential resection with end-to-end anastomosis which appears to be the best technique. According to Nakao and Fortner, the defect of the portal venous system is repaired by pushing the base of the small bowel mesentery upwards and achieving an end-to-end anastomosis of the superior mesenteric vein to the portal vein [18, 19].

If the defect overreaches 7 cm or when mobilization of the base bowel is insufficient or not achievable, Smoot's technique using left renal vein should be preferred [20]. This technique allows staying in the same operative field; moreover, caliber and thickness of the venous renal wall are similar to those of the portal vein. According to Mitsuta's study, reimplantation of the SV must take into account the direction of its flow [21].

In conclusion, after portal vein resection achieved in the course of a PD, a lateral resection and reconstruction with venorrhaphy should be chosen only when located 1 cm above the splenomesenteric confluent. When resection of confluent is required, circumferential resection with end-to-end anastomosis should be achieved. If this type of reconstruction appears to be impossible, the use of a PTFE graft must be avoided and the left renal vein can be a warranty used to carry out the venous reconstruction. Whatever might be the technical choice of venous reconstruction, curative postoperative anticoagulation does not prevent efficiently the onset of thrombosis.

## Figures and Tables

**Table 1:** Types of resection and anticoagulation treatment associated with complications (hemorrhagic, thrombosis) and mortality rates.

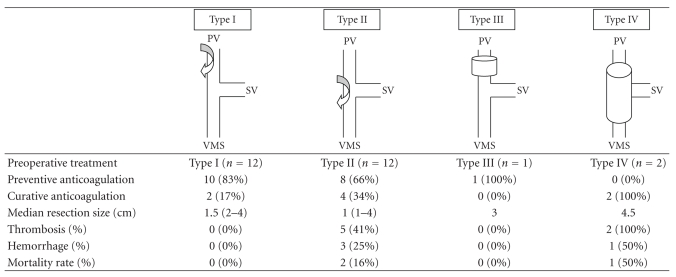

**Table 2:** Morbidity and mortality for all patients with venous resection.

	Group
Postoperative stay (days)	19 ± 9
Postoperative in intensive care unit stay (days)	3 ± 4
Postoperative death	3 (11%)
Overall number of patients with complications	13 (48%)
Major surgical complications	8 (30%)
Thrombosis rate	7 (26%)
Hemorrhagic rate	4 (15%)
Reoperation	4 (15%)
Minor surgical complications	5 (18.5%)
Gastric atony	5 (18.5%)
Medical morbidity	1 (4%)
Embolism pulmonary	1 (4%)
Blood transfusion	16 (57%)
